# Insights into durability against resistance from the antibiotic nitrofurantoin

**DOI:** 10.1038/s44259-024-00056-1

**Published:** 2024-11-25

**Authors:** Riannah Kettlewell, Charlotte Jones, Timothy W. Felton, Mato Lagator, Danna R. Gifford

**Affiliations:** 1 Division of Evolution, Infection & Genomics, School of Biological Sciences, Faculty of Biology, Medicine and Health, The University of Manchester, Manchester, UK; 2 Division of Immunology, Immunity to Infection & Respiratory Medicine, School of Biological Sciences, Faculty of Biology, Medicine and Health, The University of Manchester, Manchester, UK; 3 Wythenshawe Hospital, Manchester University NHS Foundation Trust, Manchester, UK

**Keywords:** Antimicrobial resistance, Molecular evolution, Drug regulation

## Abstract

Nitrofurantoin has shown exceptional durability against resistance over 70 years of use. This longevity stems from factors such as rapid achievement of therapeutic concentrations, multiple physiological targets against bacteria, low risk of horizontal gene transfer, and the need to acquire multiple mutations to achieve resistance. These combined features limit resistance emergence and spread of nitrofurantoin resistance. We propose nitrofurantoin as an exemplar for developing other durable treatments.

The escalating spread of antimicrobial resistance poses a considerable challenge to global public health. For the majority of antibiotics, resistance usually emerges within a decade of its first clinical use^1^. However, nitrofurantoin stands out as an exception to this trend, as rates of resistance have remained low, despite high frequency of usage as a treatment for urinary tract infections (UTIs)^2^. Lessons learned from nitrofurantoin’s robustness could prove useful in the attempt to develop other antibiotic treatment strategies that are durable against resistance.

Nitrofurantoin is a synthetic antibiotic, originally patented in 1952 by Eaton Laboratories in Norwich, New York, USA. It first entered clinical use in 1953 and was marketed under the brand names Furadantin, Macrobid, and Macrodantin^3^. Though its mechanism of action was not completely understood at the time, it was known to affect metabolism through inhibition of bacterial enzymes and to interfere with cell wall synthesis, among other effects. Nitrofurantoin’s broad-spectrum activity against both gram-negative and gram-positive bacteria, including common uropathogens *Escherichia coli, Klebsiella* spp., *Enterococcus* spp., and *Staphylococcus* spp., made it particularly effective, and it quickly became widely used for the treatment of uncomplicated UTIs^4^. However, some less common uropathogens, including *Pseudomonas aeruginosa* and *Proteus* spp., were recognised to be intrinsically resistant^5^.

Today nitrofurantoin is one of the most prescribed treatments for UTIs. Nitrofurantoin is included on the World Health Organization’s List of Essential Medicines^6^, and its empirical use is encouraged through inclusion in the Access group of The WHO AWaRe antibiotic book^7^. In 2016, English prescribing guidelines switched from trimethoprim to nitrofurantoin as the first-choice antibiotic for the treatment of lower urinary tract infections in the community context^8^. Prescription data in England shows a steady rise in nitrofurantoin prescription, climbing from 35.3% in 2016 to 73.1% in 2023, coupled with a reduction in trimethoprim use (Fig. 1A)^9^. In real terms, the average number of nitrofurantoin prescriptions effectively doubled between 2015 and 2020 (2.1 vs. 4.2 million prescriptions/year), remaining relatively constant since. Over the same period, there has been a slow but steady decline in the frequency of *E. coli* isolates with trimethoprim resistance without a corresponding increase in nitrofurantoin resistance, which has hovered around 2.5% for *E. coli* and coliform isolates sampled from community populations (Fig. 1B). A statistical model relating resistance to antibiotic, date and their interaction is a good fit to the data (overall fit of linear regression: adjusted *R*^2^ = 0.98, *F*_3,68_ = 1307, *p* < 0.0001). There is only a significant relationship with date for trimethoprim resistance (interaction effect = −1.8 × 10^−5^ ± 0.37 × 10^−5^ S.E., *t*_1_ = −4.9, *p* < 0.00001).

**Fig. 1:**
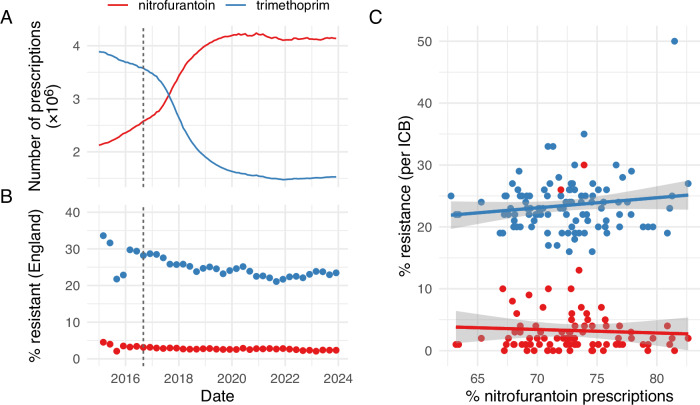
Increased nitrofurantoin prescribing is not associated with increased nitrofurantoin resistance (twelve-month rolling averages, data from England, 2015—2023). **A** Count of nitrofurantoin and trimethoprim prescriptions. **B** Resistance to nitrofurantoin and trimethoprim in *E. coli* and other coliforms isolated from urine. Vertical dashed lines in **A** and **B** indicate date of switch to nitrofurantoin as first-choice antibiotic. **C** Resistance and nitrofurantoin prescribing for each of the 42 healthcare regions in England (Integrated Care Boards, ICBs), averaged across the twelve-month period ending in December 2023 (grey areas denote 95% confidence interval for the linear regression; clipped symbols at the bottom of the plot denote ICBs with 0% recorded resistance). Data from the UK Office for Health Improvement and Disparities Fingertips data collection^9^.

While there is variation within geographic regions in the UK for nitrofurantoin prescribing (62.7–82.6%, relative to trimethoprim prescription) and resistance (0–30%), there is little relationship between the two. A statistical model relating nitrofurantoin resistance to prescribing rates does not describe the data well (overall fit of linear regression: adjusted *R*^2^ = 0.01, *F*_1,85_ = 1.8, *p* = 0.18), and there is no significant association between nitrofurantoin prescribing and resistance (slope = −2.2 × 10^−5^ ± 1.6 × 10^−5^ S.E., *t*_1_ = −1.35, *p* = 0.18, Fig. 1C). Similar nitrofurantoin resistance rates and patterns are seen globally^10–12^, though are on average higher in the United States (>20%^13^). This is in stark contrast to many other antibiotics, where rates of prescription or use correlate, often strongly, with resistance rates^14–17^.

An antibiotic with enduringly low rates of resistance amid increasing rates of prescription is of great interest in the fight against antimicrobial resistance. Understanding the mechanisms behind the surprising success of nitrofurantoin could help preserve it as an effective treatment and also aid in the development of new antibiotic compounds or treatment approaches that minimise resistance. We argue that several aspects of nitrofurantoin contribute to its durability against resistance evolution.

## Properties of nitrofurantoin contributing to its durability against resistance

### Rapid achievement of therapeutic concentrations specific to the site of infection

The favourable pharmacokinetic and pharmacodynamic (PK/PD) properties of nitrofurantoin treatment likely aid the suppression of resistance evolution^18–22^, as resistance has been experimentally demonstrated to evolve faster at low concentrations^23^. High therapeutic concentrations of nitrofurantoin are rapidly achieved, and specific to the site of infection in the urinary tract. After oral administration, nitrofurantoin is rapidly excreted unchanged in the urine, achieving concentrations of over 200 μg/ml^3^—well above the mutant prevention concentration of 64 μg/ml^24^. This allows nitrofurantoin to effectively kill bacteria in the urinary tract before the emergence of de novo-resistant mutants. Exploiting collateral sensitivity could further enhance killing, as mutants resistant to several antibiotics have increased susceptibility to nitrofurantoin^25,26^. In contrast, antibiotics that do not rapidly achieve high localised concentrations at the site of infection are more vulnerable to the development of resistance, as susceptible strains can proliferate and accumulate resistance mutations before the antibiotic reaches inhibitory concentrations. The rapid increase in concentrations impedes evolution particularly well when clinical resistance to a drug requires stepwise accumulation of multiple mutations—as is the case for nitrofurantoin^27^.

### Low off-target concentrations

Nitrofurantoin rapidly achieves therapeutic concentrations at the site of infection (i.e. the bladder), with low off-target concentrations. While it achieves concentrations of over 200 μg/ml in urine^3^, concentrations in other bodily fluids are much lower; for example, typical plasma levels are less than 1 μg/ml, which is below the concentration needed to select for antimicrobial resistance mutations^24^. Nitrofurantoin is rapidly absorbed from the small intestine^20^, with only 2% of the dose recovered in faeces in a rat model^28^. This low concentration in the gut reduces the selection pressure for resistance within the microbiome, which can otherwise serve as a reservoir for resistant strains^29^. As a result, nitrofurantoin-resistant organisms are only rarely isolated from faeces^30,31^. Nitrofurantoin also does not cause dysbiosis of the normal intestinal microbiome^32,33^, a state that can result in increased colonisation by pathogenic organisms.

### Multifactorial mechanism of action on bacterial physiology

An aspect of nitrofurantoin’s robustness against antibiotic resistance development likely stems from its multifactorial mechanism of action. Nitrofurantoin itself is not directly bactericidal; its activity requires the reduction of the nitro group in its molecular structure by bacterial nitroreductases after uptake into the cell^34^. This generates reactive intermediates that target multiple vital metabolic processes, such as protein synthesis, the citric acid cycle, and DNA/RNA synthesis^3,35^. These multifactorial effects within bacteria make resistance development highly complex and difficult to achieve, as changes (through mutations) to multiple mechanisms are required to concurrently overcome each damaging effect^3^.

### Requirement for multiple independent mutations for clinical resistance

As nitrofurantoin’s mechanism of action involves several different bacterial nitroreductases, including NfsA and NfsB^36^, de novo resistance emergence requires changes to multiple targets. Separate inactivating mutations in both *nfsA* and *nfsB* are typically required for clinical levels of resistance and, indeed, are observed in clinically resistant organisms^24,37,38^. Crucially, the inactivation of just one out of *nfsA* and *nfsB* confers only a small increase in the minimum inhibitory concentration (MIC)^24^. Coupled with the favourable PK/PD properties of nitrofurantoin, this reduces the probability of resistance emerging via stepwise loss of *nfsA* and *nfsB*.

A single large deletion mutation that simultaneously inactivates both *nfsA* and *nfsB* is likewise an unlikely route to acquiring resistance. Despite being functionally similar, *nfsA* and *nfsB* are located far apart within the genome. In the type strain *E. coli* str. K-12, the distance between the two genes is 287 kbp^24^ (ranging between 262–337 kbp in other isolates); this genomic region contains 260 genes, of which 16 are identified as essential^39^. A genomic deletion inactivating both simultaneous genes would, therefore, likely be non-viable.

The proper functioning of nitroreductases is also dependent on other genes, including *ribE*, which is required to produce co-factors used by NfsA and NfsB. Inactivating mutations in *ribE* can also give rise to some degree of resistance^37,40^, but frequencies of *ribE* mutations among nitrofurantoin-resistant clinical isolates are low and are not found without *nfsA* and *nfsB* mutations^36^, suggesting other factors may constrain its contribution to resistance in vivo. Notably, intrinsically resistant organisms inherently lack the specific nitroreductases that convert nitrofurantoin into its active form^5^.

### Fitness costs associated with resistance mutations

Fitness costs imposed by resistance mutations in the absence of antibiotics may contribute to an antibiotic’s durability. When antibiotic treatment is withdrawn, resistant organisms have a reduced ability to disseminate and establish infections. While nitrofurantoin resistance has been shown to impose a fitness cost, the extent and significance of this cost are debated. A recent study on clinical isolates found a 2–10% reduction in growth rate, though the results were not statistically significant^24^. An earlier study reported an average 6% reduction, with some isolates experiencing considerably higher costs^41^. Experimental studies have demonstrated up to a 10% reduction in competitive fitness, though this cost was able to be mitigated through compensatory adaptation^42^. However, it is important to note that these fitness costs were measured in vitro. To fully understand the implications of resistance, fitness costs should be assessed in vivo, where environmental and host factors may influence outcomes.

### Resistance typically emerges due to mutations rather than horizontal gene transfer

For many antibiotics where resistance has become common, horizontal gene transfer (HGT) is the major route to resistance acquisition. This often involves antibiotic-inactivating enzymes, target bypass genes, or novel efflux pumps^43^. Such genes are often carried on mobile genetic elements like plasmids or integrons, or acquired through natural transformation, all of which facilitate spread between species^44,45^. Unlike many other antibiotics in common use, the first two resistance mechanisms play only a small part in nitrofurantoin resistance. There are limited reports of nitrofurantoin-inactivating enzymes in clinical isolates^46^, although the presence of nitrofurantoin-degrading enzymes in natural isolates suggests that horizontal gene transfer could eventually allow pathogens to acquire these resistance mechanisms^47^. Target bypass is unlikely to result in nitrofurantoin resistance, given that its mechanism of action does not rely on inhibiting a specific gene target. Recombination is also unlikely to transfer resistance because, as previously mentioned, the genomic region between the *nfsA* and *nfsB* is large^24^.

In contrast, the HGT of a novel multi-drug efflux pump, OqxAB, has been linked to nitrofurantoin resistance^48^. OqxAB was first detected in *E. coli* from pigs from Denmark^49,50^, and is associated with the feed additive olaquindox, which had been in use from the 1980s until an EU-wide ban in 1999. *oqxAB* was found to be carried on a conjugative plasmid (the IncX1-type plasmid pOLA52^51^), which has facilitated its spread between different bacteria. *oqxAB* is now carried by a variety of mobile genetic elements capable of transmitting between strains. The prevalence of *oqxAB* can exceed 50% in animal isolates from regions where olaquindox remains in use (including Australia^52^ and China^53,54^). In human isolates, prevalence ranges from 0.4–42%^54^, but is higher in regions using olaquindox, particularly among farmworkers^55^. Though OqxAB on its own confers only a modest increase in nitrofurantoin MIC, when combined with a *nfsA* mutation, it can confer resistance above the clinical breakpoint^48^. Work in other organisms has shown that the acquisition of novel efflux pumps can increase the potential for high-level resistance to evolve by allowing additional time for resistance mutations to emerge^56^. The potential for OqxAB to act as a similar “stepping stone” to full nitrofurantoin resistance in clinical isolates should be assessed.

The reduced role of HGT in nitrofurantoin resistance may be a fortunate consequence of its synthetic origins. Being a fully synthetic compound, nitrofurantoin likely lacks a natural reservoir of resistance genes. Many antibiotics in common use are derived from compounds originally produced by microbes and may have initially evolved for microbe-microbe competition^57^. Genes for synthesising antibiotics are evolutionarily ancient^58,59^—certainly long predating anthropogenic use of antibiotics^60^, and often contemporaneous with major evolutionary events^59^. Consequently, there has been an “arms race” between the evolution of antibiotic synthesis genes and antibiotic resistance genes that have been running for millions of years^61^. Consequently, in some cases, resistance to an antibiotic is already present in bacterial populations before its introduction into commercial use^62^. The use of fully synthetic antibiotics may help reduce resistance, to a degree^63^. However, not all synthetic antibiotics are inherently durable against resistance evolution; other synthetic antibiotics, such as fluoroquinolones and trimethoprim, exhibit significantly higher resistance^64–67^.

## Insights into factors promoting nitrofurantoin resistance

Despite many properties favouring its durability against resistance evolution, nitrofurantoin resistance does emerge in both experimental and clinical contexts. Insight into how and why nitrofurantoin resistance does emerge can help ensure its continued efficacy.

While full nitrofurantoin resistance imposes a fitness cost in the absence of the drug^24,41^, the acquisition of an initial inactivating mutation in *nfsA* or *nfsB* can be selectively advantageous at sub-inhibitory nitrofurantoin concentrations, even without significantly changing the MIC^24^. Intriguingly, when resistance has been observed to arise in vitro, mutations tended to arise first in *nfsA*, followed by mutations in *nfsB*, due to the larger fitness benefits conferred by loss of *nfsA* under sub-inhibitory concentrations^24^.

Treatment noncompliance could play a significant role in contributing to nitrofurantoin resistance. As subinhibitory nitrofurantoin concentrations can facilitate resistance^23,41^, non-adherence with treatment regimens could allow these first-step mutants to proliferate and acquire second-step mutations. Ensuring consistent nitrofurantoin levels through full adherence is crucial to preventing the development of resistance. A patient-focused intervention emphasising the importance of adherence could help preserve the efficacy of nitrofurantoin, benefiting both the individual patient and the broader community by reducing the risk of resistance development and maintaining effective treatment options.

The use of antibiotics as prophylactics is a potential risk factor for increasing the prevalence of antibiotic resistance. Prophylactic use can expose bacteria to sub-therapeutic levels of antibiotics, promoting the selection and spread of resistant strains^68^. Existing evidence suggests the risk of nitrofurantoin resistance developing during prophylaxis is relatively low. A meta-analysis of controlled trials has shown that rates of nitrofurantoin resistance reported during trials were low^69^. In one study, nitrofurantoin prophylaxis did not increase resistance in children with urinary tract abnormalities^70^. However, a second study observed that *E. coli* was often replaced by resistant *Klebsiella* spp. and (intrinsically) resistant *Pseudomonas* spp^71^. Low-dose prophylaxis in adult women has generally shown low resistance rates^72,73^, although one study found nitrofurantoin prophylaxis increased resistance frequency relative to control (24% vs. 9%^74^). Generally, nitrofurantoin had lower resistance rates than alternative antibiotics^72–74^. While current evidence suggests prophylaxis poses a low risk for nitrofurantoin resistance, continued surveillance is recommended, given the emergence of new resistance mechanisms^46,48,49,54^.

A problematic development is the rise of nitrofurantoin resistance alongside multi-drug resistant (MDR) and extensively-drug resistant (XDR) isolates, particularly among the carbapenem-resistant *Enterobacteriaceae* (CRE). High-level nitrofurantoin resistance in CRE presents significant clinical challenges, complicating the treatment of UTIs. Nitrofurantoin-resistant *E. coli* isolates from the UK are more likely to also be MDR or XDR, compared to susceptible isolates^75^. Nitrofurantoin resistance is also found in non-*E. coli* CRE, including *Klebsiella pneumoniae*, *Enterobacter* spp. (including *E. cloacae*), *Citrobacter freundii* and others^37,76^. In addition to typical mutations in *nfsA* and *nfsB*, overexpression of efflux pumps^37,77^, and reduced drug uptake due to disruption of outer membrane porins^77^ likely contribute to nitrofurantoin resistance in CRE. The association between nitrofurantoin resistance and CRE is especially concerning, as nitrofurantoin has been reintroduced alongside carbapenems and colistin to counter multi-drug resistant infections^76^.

Other genomic and environmental factors affecting the evolvability of nitrofurantoin resistance warrant consideration. Recent experimental work has shown that “mutators” with defects in DNA replication fidelity can more easily acquire stepwise resistance mutations^78,79^ and overcome fitness deficits through compensatory adaptation^80^. Mutators pose a specific risk for nitrofurantoin resistance, increasing both the probability of resistance evolution and the specificity of resistance mutations^81^. The increasing prevalence of OqxAB efflux pumps also provides a potential mechanism that may make high-level nitrofurantoin resistance easier to evolve. The case of OqxAB, which was selected for in response to a different compound, also emphasises the need to better test for potential cross-resistance between existing and new drugs and chemicals prior to their release on the market.

Despite an increasing frequency of usage, nitrofurantoin has remained a durable antibiotic option for treating urinary tract infections. This durability is likely due to a few key factors, including targeted delivery to the site of infection and favourable PK/PD properties, multitarget effects on bacterial physiology, a requirement for multiple independent mutations for resistance to be acquired, fitness costs associated with resistance in the absence of nitrofurantoin, and the near-absence of horizontally acquired resistance. Continued judicious use of nitrofurantoin will hopefully continue to preserve this important antibiotic option for treating UTIs in the face of increasing antimicrobial resistance. However, more insight needs to be gained about factors that can increase the evolvability of nitrofurantoin resistance, including treatment non-adherence, the use of nitrofurantoin as a prophylactic treatment for recurrent UTI, bacterial mutation rate evolution, and the horizontal acquisition of novel efflux pumps. More generally, insight into mechanisms favouring the durability of nitrofurantoin may prove useful in the development of other durable antibiotics and treatment options.

## Supplementary information

Supplementary File S1

## Data Availability

The datasets used are from the AMR Local Indicators datasets produced by the UK Health Security Agency (UKHSA), which can be accessed via the UK Office for Health Improvement and Disparities Fingertips public health data portal (https://fingertips.phe.org.uk/). Prescribing data is taken from indicator ID 92511. Data on nitrofurantoin resistance in urine specimens is taken from indicator ID 92521. Data on trimethoprim resistance in urine specimens is taken from indicator ID 92519. The data were analysed in R using a custom script, provided as Supplementary File S1.
